# Vascular conductance and muscle blood flow during exercise are altered by inspired oxygen fraction and arterial perfusion pressure

**DOI:** 10.14814/phy2.13144

**Published:** 2017-03-14

**Authors:** Rodrigo Villar, Richard L. Hughson

**Affiliations:** ^1^ Division of Natural SciencesFaculty of Health SciencesFranklin Pierce UniversityRindgeNew Hampshire; ^2^ Faculty of Applied Health SciencesUniversity of WaterlooWaterlooOntarioCanada; ^3^ Coordenação de Aperfeiçoamento de Pessoal de Nivel Superior (CAPES)Brasilia DFBrazil; ^4^ Schlegel‐University of Waterloo Research Institute for AgingWaterlooOntarioCanada

**Keywords:** Doppler ultrasound, electromyography, hypoxia, maximal vasodilatory capacity, oxygen delivery

## Abstract

We tested the hypothesis during the combined challenges of altered inspired O_2_ fraction (F_I_O_2_) and posture changes at lower power output regardless of body position that the vascular conductance (VC) recruitment to the exercising muscle would not limit muscle perfusion and estimated O_2_ delivery (DO_2*est*_). However, in head‐down tilt at the higher power output exercise in hypoxia, the recruitment of VC would have a functional limitation which would restrict muscle blood flow (MBF) leading to a limitation in DO_2*est*_ with consequent increases in metabolic stress. Ten healthy volunteers repeated plantar flexion contractions at 20% (low power output = LPO) and 30% (higher power output = HPO) of their maximal voluntary contraction in horizontal (HOR), 35° head‐down‐tilt (HDT) and 45° head‐up‐tilt (HUT). Popliteal diameter and muscle blood flow velocity were measured by ultrasound determining MBF. VC was estimated by dividing MBF flow by MPP, and DO_2*est*_ was estimated by MBF times saturation. LPO_*HUT*_ in hypoxia was associated with no changes in VC and MBF leading to reduced DO_2*est*_. In LPO_*HDT*_ under hypoxia, despite no apparent functional limitation in the VC recruitment, rise in MBF to maintain DO_2*est*_ was associated with marked increase in muscle electromyographic activity, indicating greater metabolic stress. In HPO_*HDT*_ under hypoxia, a functional limitation for the recruitment of VC constrained MBF and DO_2*est*_. Elevated muscle electromyographic signal in HPO_*HDT*_ under hypoxia was consistent with challenged aerobic metabolisms which contributed to a greater increase in the relative stress of the exercise challenge and advance the onset of muscle fatigue.

## Introduction

Muscle blood flow increases to match the delivery of O_2_ to the metabolic demand during exercise with constant intensity (Laughlin and Joyner 2003; Walker et al. 2007). This matching is accomplished through a combined effect of progressive vasodilatory mechanisms and potential contribution of the muscle pump. In submaximal steady‐state exercise performed with small muscle mass, muscle blood flow determines O_2_ delivery because there is no alteration in the arterial O_2_ content (Walker et al. 2007). However, in conditions with altered inspired O_2_ fraction, arterial O_2_ content and arterial O_2_ partial pressure change and compensatory adjustments in muscle blood flow mediated by vasodilation (Ellsworth et al. 1995; Jia et al. 1996; Stamler et al. 1997; Calbet 2000) are required for regulation of O_2_ delivery (Gonzalez‐Alonso et al. 2001) to maintain muscle O_2_ uptake (Koskolou et al. 1997).

During submaximal one or two‐legged knee extension exercise as well as submaximal calf exercise in hypoxia compensatory adjustments in muscle blood flow maintained O_2_ delivery (Koskolou et al. 1997; Gonzalez‐Alonso et al. 2001; DeLorey et al. 2004; Donnelly and Green 2013); however, one study reported for submaximal alternated two‐legged kicking exercise in hypoxia there was maintenance of muscle blood flow with reduction in O_2_ delivery (MacDonald et al. 2001). Manipulations of limb position relative to the heart to alter arterial and venous pressure also have been used in normoxic conditions to investigate muscle blood flow and O_2_ delivery adaptations during exercise. Villar and Hughson (1985) and Walker et al. (2007) provided evidence that, despite greater vascular conductance, muscle blood flow during exercise was reduced with the limb positioned above the heart level. However, the effects of altered inspired O_2_ fraction (hypoxia) in conjunction with altered muscle perfusion pressure on vascular conductance, muscle blood flow, and O_2_ delivery responses during exercise in humans are not known.

The purpose of this study was to investigate vascular conductance, muscle blood flow, and O_2_ delivery responses during submaximal calf exercise in three body positions (HOR, horizontal, HDT, head‐down tilt, and HUT, head‐up tilt that affect the gravity‐dependent distribution of arterial and venous pressures at two work rates (lower and higher power outputs) under two inspired O_2_ fractions (normoxia and hypoxia). It was hypothesized that for the lower power output exercise under hypoxia in any body position, the recruitment of vascular conductance would not limit muscle perfusion and O_2_ delivery. However, at the higher power output exercise in head‐down tilt, a functional limitation to the recruitment of vascular conductance would occur in hypoxia restricting the increase in muscle blood flow leading to a limitation in O_2_ delivery with consequent increases in metabolic stress.

## Methods

### Participants

Ten healthy male volunteers from our previous study (Villar and Hughson 1985) returned to participate in this new research phase. They were not specifically exercise trained and had average physical characteristics of: age 27.2 ± 3.6 years, height 176.7 ± 5.5 cm, body mass 78.6 ± 6.9 kg, and body mass index 25.2 ± 2.5 kg/m^2^. Prior to signing an information consent form approved by the Office of Research Ethics of the University of Waterloo, participants received complete written and verbal details of the experimental procedures and potential risks involved. They were instructed to refrain from consuming caffeinated beverages, alcohol, or engaging in vigorous exercise for 24 h prior to testing, and from consuming a large meal within 2 h of testing. Tests were conducted with room temperature constant at 19.6 ± 1.0°C to ensure minimal presence of skin blood flow, humidity at 40.1 ± 4.5% and barometric pressure at 731.8 ± 2.2 mmHg.

### Experimental design

Participants reported to the laboratory five times. In random order, each participant completed tests of peak vascular conductance and four tests with changes in body position (HOR, HDT, HUT) as described elsewhere (Villar and Hughson 1985, 2013) and inspired O_2_ fraction (normoxia = 21% O_2_ and hypoxia, 14% O_2_). Briefly, after arrival in the laboratory, participants assumed a prone position to facilitate popliteal artery access. Their heads were supported by a massage table head piece, shoulder blocks were adjusted and their arms were placed with the shoulders and elbows positioned at ~90°. During testing, participants were secured by two belts, one situated on the chest, the other on the hips. Straps from these belts were connected to the tilt table to prevent sliding. A footplate was attached to the tilt table with the right foot strapped on this footplate to allow plantar flexion exercise. Manipulations of inspired O_2_ fraction (F_I_O_2_) were performed with the participants breathing room air or switching to hypoxic air through a face mask connected via a sterile air filter to a large nonpressurized bag (Nondiffusing gas collection bag 60L, Hans Rudolph Inc., Kansas City, Missouri), containing medical grade gas mixtures with 14% O_2_ and balance nitrogen from a cylinder tank (Praxair Canada Inc., Mississauga, Ontario, Canada).

The angle of tilting for head‐down tilt (HDT = 35˚) accomplished a reduction in muscle perfusion pressure (MPP, estimated to the middle of the calf muscle) of ~44 mmHg and an increase of ~55 mmHg in head‐up tilt (HUT = 45˚) (Villar and Hughson 1985, 2013). The changes from normoxia to hypoxia during exercise accomplished a reduction of ~8% in arterial oxygen saturation (pulse oximetry) from 97.2 ± 0.64% to 89.0% ± 1.2, respectively, which is consistent with previous research (DeLorey et al. 2004). The participants rested before starting the testing for ~30 min, while instrumentation took place as described below.

### Peak vascular conductance protocols

Reactive hyperemia following release of an arterial occlusion cuff plus exercise was used to obtain peak vascular conductance (VC_*peak*_) in each of the three body positions as described previously (Villar and Hughson 1985). Briefly, baseline data were collected for 1 min prior to inflation of a cuff placed around the lower leg just distal to the popliteal fossa to 300 mmHg for 2 min. Isometric plantar flexion exercise was performed at 50% maximal voluntary contraction (MVC) for 1 min during the inflation period. Peak muscle blood flow velocity, popliteal artery diameter, and local arterial blood pressure were used in calculation of peak vascular conductance.

### Altered arterial perfusion pressure and inspired O_2_ fraction exercise protocols

After 3 min of baseline data recording, participants performed a repeated plantar flexion exercise (3 sec duty cycle, 1 sec contraction, 1 sec to lower load, 1 sec rest) with contractions of ~20% MVC (lower power output, 3.0 ± 0.4 kg, 2.0 ± 0.2 W) and ~30% MVC (higher power output, 6.0 ± 0.7 kg, 4.0 ± 0.5 W) for 7 min (3 min in normoxia and 4 min in hypoxia) followed by 3 min of recovery in hypoxia. To isolate contractions of the calf muscles, the foot plate movement was set in the rotational axis of the ankle with the angle of movement at ~20° representing a displacement of 10 cm with minimal heel lift. The weights attached to the footplate were altered upon changing the angle of the tilt table to obtain the same cable tension in all three positions. In HDT, the load was reduced by 0.6 kg, whereas the load in HUT was increased by 0.8 kg. The monitoring of muscle activity was used to confirm the equality of work rates (Villar and Hughson 1985). A light system installed on the custom‐built tilt table was used to give visual feedback to the participants to ensure full range of motion.

Six different protocols were performed for each participant; three tests per day on four different days randomized by blocks and counterbalanced among subjects allowing each protocol to be performed twice. The four days of testing were performed two times per week separated by at least 48 h. Participants were allowed to come off the tilt table and rest prior to the next test bout between each individual test on each day. The order for test block A was: higher power output exercise in head‐up tilt (HPO_*HUT*_), lower power output exercise in head‐down tilt (LPO_*HDT*_) and higher power output exercise in horizontal (HPO_*HOR*_). The order for test block B was: lower power output exercise in horizontal (LPO_*HOR*_), higher power output exercise in head‐down tilt (HPO_*HDT*_) and lower power output exercise in head‐up tilt (LPO_*HUT*_).

### Data acquisition

Muscle blood flow (MBF) measured from the popliteal artery was calculated from mean flow velocity (4 MHz pulsed Doppler, Neurovision Doppler Ultrasound, Model 500; Multigon Industries, Mt. Vernon) and diameter (8–12 MHz linear probe, M5 Diagnostic Ultrasound system, Mindray Bio‐medical electronics, Shenzen, China) was used to record popliteal arterial diameter (AD_*pop*_) in B‐mode. Three separate AD_*pop*_ measurements obtained during diastole were averaged in baseline, at the end of recovery, and during the relaxation phase between contractions in the third (normoxia) and the sixth (hypoxia) minute of exercise. Measurements and calculations for MBF and vascular conductance (VC) have low variability, consistency, and good agreement in the conditions of our laboratory during testing in normoxia and hypoxia as reported previously (Villar and Hughson 2013).

Continuous finger arterial blood pressure (Finometer, Finapres Medical Systems, Arnhem, the Netherlands), RR‐interval obtained from an electrocardiogram (Pilot 9200, Colin Medical Instruments Corp, San Antonio, Texas), and arterial O_2_ saturation acquired by a pulse oximeter with the probe positioned in the index finger of the left hand (Ohmeda 3740 Pulse oximeter, Louisville, CO.) were digitally recorded (Powerlab, ADInstruments, Colorado Springs) at 1000 Hz. The muscle perfusion pressure (mmHg) was estimated by mean arterial pressure (mmHg) and the distance between subject's heart and the middle of the calf muscle for all body positions converted to millimeters of mercury (1 mmHg = 1.36 cm of H_2_O) due to the effects of gravity on arterial perfusion pressure.

Electromyography (EMG) used skin surface disposable electrodes (Blue Sensor, Medicost, Inc., Olstykke, Denmark) to measure muscle activity from the right leg on the distal half of the medial gastrocnemius, lateral gastrocnemius, and soleus muscles as described previously (Villar and Hughson 1985). The raw EMG signal was amplified by a custom built amplifier (bandwidth 20–500 Hz, common mode rejection rate >90 db, input impedance 2 MΩ) and processed with custom software (Matlab, version 7.0; The Mathworks, Natick, MA).

### Data analysis

#### Vascular conductance

Changes in vascular conductance (VC) during exercise were measured in absolute values as well as being expressed relative to the peak vascular conductance after occlusion plus exercise. VC was calculated over each complete 3 sec cycle including: contraction, lowering load, and relaxation. As well, the relaxation VC (VC_*relax*_) was determined using only VC during the relaxation phase to assess the magnitude of vasodilation.

#### Altered arterial perfusion pressure and inspired O_2_ fraction exercise

Beat‐by‐beat data were time aligned, linearly interpolated and averaged over two complete contraction/relaxation cycles. VC, MBF, estimated O_2_ delivery, and EMG were determined in the last minute of exercise in both normoxia and hypoxia. O_2_ delivery (DO_*2est*_) was estimated as the product between muscle blood flow and arterial O_2_ saturation (DO_2*est*_ = MBF ^ ^x SaO_2_).

The EMG signal was rectified and filtered, and the integrated EMG (iEMG) was calculated from the area under the rectified signal for each contraction with mean power frequency (MPF) estimated by the fast Fourier transformation (FFT) analysis. The iEMG was normalized by the maximal isometric voluntary contraction (MVC, 61.0 ± 3.2 kg) expressed as the percentage of MVC averaged in the last minute of exercise (Villar and Hughson 1985). EMG activity during normoxia and hypoxia was obtained by the sum of the mean for soleus and gastrocnemius muscle group activity divided by the sum of the MVC times 100 and used to determine the triceps surae muscle activity (TSMA).

### Statistical analysis

Responses to the VC_*peak*_ tests were analyzed by one‐way analysis of variance for repeated measures with respect to body position. Variables measured during each of the constant load exercise tests in different positions were assessed over the last minute of exercise at each inspired gas level by a two‐way repeated measures analysis of variance with main effects of body position and F_I_O_2_ (normoxia and hypoxia). Variables measured during baseline and the last minute of recovery at each body position underwent a two‐way analysis of variance for repeated measures with main effects of body position and time (baseline and recovery). The Student–Newman–Keuls post hoc test was performed to identify the statistically significant main effects and interactions. The level of significance was set at *P* < 0.05. Data were presented as means ± standard deviation (SD).

## Results

### Peak vascular responses

There were main effects of body position for each peak muscle blood flow velocity, peak muscle blood flow, and local arterial blood pressure (*P* < 0.05). The post hoc analyses evidenced that these variables were significantly different across all conditions, with higher values in HUT and lower in HDT than HOR (*P* < 0.05). However, VC_*peak*_ and AD_*pop*_ were not significantly different in any of the three body positions.

### Vascular responses during lower and higher power outputs under altered inspired O_2_ fraction

Differences in MBF at lower and higher power outputs were indicated by statistically significant main effects of body position and altered F_I_O_2_, and interaction between body position and altered F_I_O_2_. The post hoc analysis indicated that at lower power output, MBF was higher in LPO_*HOR*_ than LPO_*HDT*_ and LPO_*HUT*_ in both normoxia and hypoxia (*P* < 0.05). MBF was not different between LPO_*HUT*_ and LPO_*HDT*_ in normoxia, however, in hypoxia, MBF was lower in LPO_*HUT*_ than LPO_*HOR*_ and LPO_*HDT*_ (*P* < 0.05, Table 1). From normoxia to hypoxia at lower power output, MBF was not different within LPO_*HOR*_ and LPO_*HUT*_, but was higher in hypoxia than normoxia within LPO_*HDT*_ (*P* < 0.05, Table 1). At higher power output, MBF was higher in HPO_*HOR*_ compared to HPO_*HDT*_ (*P* < 0.05), but MBF was not different between HPO_*HOR*_ and HPO_*HUT*_ and between HPO_*HUT*_ and HPO_*HDT*_ in both normoxia and hypoxia (Table 1). From normoxia to hypoxia at higher power output, MBF was higher in hypoxia than normoxia within HPO_*HOR*_ and HPO_*HDT*_ (*P* < 0.05, Table 1), but was not different in HPO_*HUT*_.

**Table 1:** Muscle blood flow, estimated O_2_ delivery, and vascular conductance during lower and higher power outputs under altered muscle perfusion pressure (HOR, HDT, and HUT) and inspired O_2_ fraction (normoxia and hypoxia)

		Conditions					
Variables		LPO_*HDT*_ (*n* = 9)	LPO_*HOR*_ (*n* = 10)	LPO_*HUT*_ (*n* = 10)	HPO_*HDT*_ (*n* = 9)	HPO_*HOR*_ (*n* = 10)	HPO_*HUT*_ (*n* = 10)
MBF (mL·min^−1^)	Normoxia	146.3 ± 34.2^a^	188.7 ± 67.1	127.2 ± 58.8^a^	186.8 ± 55.4^a^	227.5 ± 79.7	203.3 ± 74.3
	Hypoxia	167.3 ± 38.1^a^^,^ c	195.8 ± 72.4	119.5 ± 53.2^a^^,^ b	221.6 ± 68.7^a^^,^ c	249.5 ± 95.7^c^	210.3 ± 84.2
DO_*2est*_ (mLO_2_·min^−1^)	Normoxia	142.2 ± 33.2^a^	183.4 ± 65.6	123.9 ± 57.2^a^	180.9 ± 53.3^a^	221.0 ± 77.3	197.0 ± 72.4
	Hypoxia	152.6 ± 34.3^a^^,^ c	176.9 ± 65.4	109.6 ± 48.5^a^^,^ b ^,^ c	201.0 ± 60.8^a^^,^ c	228.4 ± 86.7	190.8 ± 75.0
VC (mL·min^−1^·mmHg^−1^)	Normoxia	2.9 ± 0.8^a^	2.2 ± 0.7	0.9 ± 0.4^a^, ^b^	3.6 ± 1.0^a^	2.4 ± 0.8	1.4 ± 0.5^a^^,^ b
	Hypoxia	3.3 ± 0.8^a^, ^c^	2.1 ± 0.8	0.8 ± 0.4^a^, ^b^	4.1 ± 1.2^a^, ^c^	2.5 ± 0.9	1.4 ± 0.6^a^^,^ b
Test block Order		A2	B1	B3	B2	A3	A1

Values are mean ± SD. *n,* Number of participants ; MBF, muscle blood flow; DO_2est,_ estimated O_2_ delivery, VC, vascular conductance; LPO_*HOR,*_ lower power output in horizontal; LPO_*HDT*_, lower power output in head‐down tilt; LPO_*HUT*_, lower power output in head‐up tilt; HPO_*HOR,*_ higher power output in horizontal; HPO_*HDT,*_ higher power output in head‐down tilt; HPO_*HUT*_, higher power output in head‐up tilt. HDT, head‐down‐tilt; Test Block Order indicates for each of the Blocks.

a Significant differences compared with HOR.

b HDT within the same gas condition and experimental phase.

c Significant differences compared with normoxia within the same body position (*P* < 0.05).

For DO_2*est*_ at lower and higher power outputs, there were significant main effects of body position and altered F_I_O_2_, and interaction between body position and altered F_I_O_2_. The post hoc analysis indicated that at lower power output, DO_2*est*_ was higher in LPO_*HOR*_ than LPO_*HDT*_ and LPO_*HUT*_ in both normoxia and hypoxia (*P* < 0.05). DO_2*est*_ was not different between LPO_*HUT*_ and LPO_*HDT*_ in normoxia, however, in hypoxia, DO_2*est*_ was lower in LPO_*HUT*_ than LPO_*HOR*_ and LPO_*HDT*_ (*P* < 0.05, Table 1). From normoxia to hypoxia at lower power output, DO_2*est*_ was not different within LPO_*HOR*_, but was lower within LPO_*HUT*_ and higher within LPO_*HDT*_ (*P* < 0.05, Table 1). At higher power output, DO_2*est*_ was higher in HPO_*HOR*_ compared to HPO_*HDT*_ in both normoxia and hypoxia (*P* < 0.05, Table 1). From normoxia to hypoxia at higher power output, DO_2*est*_ was not different within HPO_*HUT*_ and HPO_*HOR*_, but was higher in hypoxia than normoxia within HPO_*HDT*_ (*P* < 0.05, Table 1).

The VC at each of the lower and higher power outputs was significantly different for main effects of body position and altered F_I_O_2_, and interaction between body position and altered F_I_O_2_. The post hoc analysis indicated that at the lower power output, VC was higher in LPO_*HDT*_, but lower in LPO_*HUT*_ in both normoxia and hypoxia compared to LPO_HOR_ (*P* < 0.05, Table 1). From normoxia to hypoxia at lower power output, VC was not different within LPO_*HOR*_ and LPO_*HUT*_, but was higher in hypoxia than normoxia within LPO_*HDT*_ (*P* < 0.05, Table 1). At higher power output, VC was higher in HPO_*HDT*_, but lower in HPO_*HUT*_ in normoxia and hypoxia (*P* < 0.05, Table 1). From normoxia to hypoxia at higher power output, VC was not different within HPO_*HOR*_ and HPO_*HUT*_, but was higher in hypoxia than normoxia within HPO_*HDT*_ (*P* < 0.05, Table 1). There were no statistically significant differences in AD_*pop*_ for all experimental conditions (data not shown).

**Figure 1:**
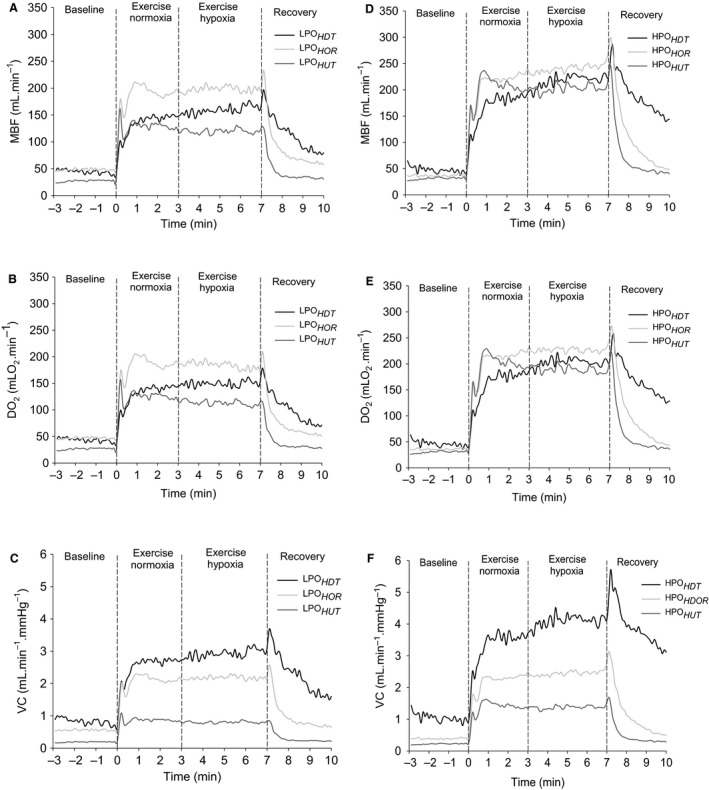
MBF (A and D), estimated O_2_ delivery (DO_2*est*_; B and E), and VC (C and F) during dynamic plantar flexion exercise performed in lower (LPO; A, B, and C) and HPO(D, E and F). Lines indicate group response and dashed vertical lines indicate the start, gas concentration switch from normoxia to hypoxia, and cessation of exercise. Data are the mean analyzed over 6‐sec time bins, including contraction and relaxation phases of the duty cycles. SD was omitted to improve data visualization. HDT, head‐down tilt; HOR, horizontal; HUT, head‐up tilt; MBF, Muscle blood flow; VC, vascular conductance; HPO, higher power outputs.

At the cessation of exercise, there were overshoots of muscle blood flow, DO_2*est*_, and vascular conductance in LPO_*HDT*_, LPH_*HOR*_, HPO_*HDT*_ and HPO_*HOR*_ (Fig. 1). The two‐way repeated‐measures ANOVA showed that by the end of the recovery time, baseline values were achieved in all but LPO_*HDT*_ and HPO_*HDT*_ as shown by the post hoc analysis (*P* < 0.05).

### Effective vascular conductance

**Figure 2:**
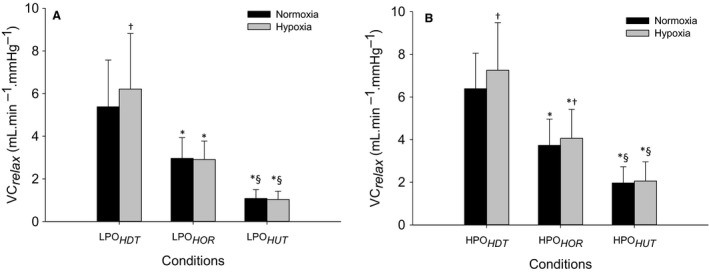
Effective vascular conductance measured during the relaxation phase of the duty cycle (VC_*relax*,_ mL·min^−1^·mmHg^−1^, left axis) and as percentage of peak VC (VC_*relax*_ %, right axis) during dynamic plantar flexion exercise. The lower (A) and higher power outputs (B) exercises were performed in HOR, HDT and HUT positions under normoxia (black) and hypoxia (gray). Data are the mean ± SD. *Statistically significant differences compared with HOR and ^§^ HDT within the same gas condition and experimental phase and ^†^statistically significant differences compared with normoxia within the same body position. HDT, head‐down tilt; HOR, horizontal; HUT, head‐up tilt.

For effective vascular conductance (VC_*relax*_) and effective vascular conductance expressed relative to the VC_*peak*_ (%VC_*relax*_) at lower and higher power outputs, there were statistically significant main effects of body position and F_I_O_2_ and interaction between body position and F_I_O_2_ as evidenced by the two‐way repeated‐measures ANOVA. The post hoc analysis indicated that at lower power output, VC_*relax*_ and %VC_*relax*_ were higher in LPO_*HDT*_ (*P* < 0.05), but lower in LPO_*HUT*_ than LPO_*HOR*_ in both normoxia and hypoxia (*P* < 0.05, Fig. 2A). From normoxia to hypoxia at lower power output, VC_*relax*_ and %VC_*relax*_ were higher in hypoxia than normoxia within LPO_*HDT*_ (*P* < 0.05), but not different within LPO_*HOR*_ and LPO_*HUT*_ (Fig. 2A). At higher power output, VC_*relax*_ and %VC_*relax*_ were higher in HPO_*HDT*_ (*P* < 0.05), but lower in HPO_*HUT*_ in both normoxia and hypoxia (*P* < 0.05, Fig. 2B). VC_*relax*_ and %VC_*relax*_ were higher in hypoxia than normoxia within HPO_*HDT*_ and HPO_*HOR*_ (*P* < 0.05) with no differences within HPO_*HUT*_ (Fig. 2B).

### Muscle activity responses under altered inspired O_2_ fraction

**Figure 3:**
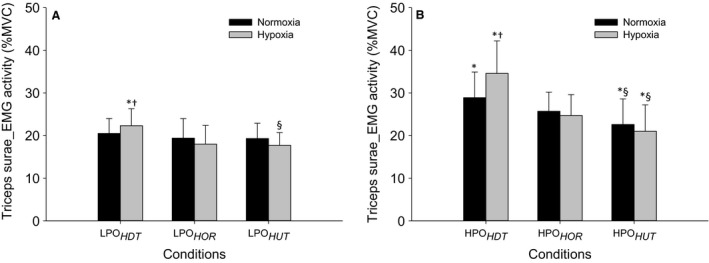
Triceps surae EMG activity during plantar flexion exercise in lower power output (A) and higher power outputs (B). Values are the mean ± SD. See Figure 1 for symbols and abbreviations.

For triceps surae muscle activity (TSMA) at lower and higher power outputs, there were significant main effects of body position and interactions between body position and F_I_O_2_ as indicated by the two‐way repeated‐measures ANOVA. The post hoc analysis indicated that at lower power output, TSMA in normoxia was not significantly different between body positions. However, in hypoxia, TSMA was higher in LPO_*HDT*_ than LPO_*HOR*_ and LPO_*HUT*_ (*P* < 0.05) with no differences between LPO_*HUT*_ and LPO_*HOR*_ (Fig. 3A). From normoxia to hypoxia at lower power output, TSMA was higher in hypoxia than normoxia within LPO_*HDT*_ (*P* < 0.05), but not different within LPO_*HOR*_ and LPO_*HUT*_ (Fig. 3A). At higher power output, TSMA was higher in HPO_*HDT*_ than HPO_*HOR*_ and HPO_*HUT*_ (*P* < 0.05), and lower in HPO_*HUT*_ than HPO_*HOR*_ in both normoxia and hypoxia (*P* < 0.05, Fig. 3B). From normoxia to hypoxia at higher power output, TSMA was higher in hypoxia than normoxia within HPO_*HDT*_ (*P* < 0.05), but not different within HPO_*HOR*_ and HPO_*HUT*_ (Fig. 3B).

### Normalized muscle blood flow to triceps surae muscle activity

**Figure 4:**
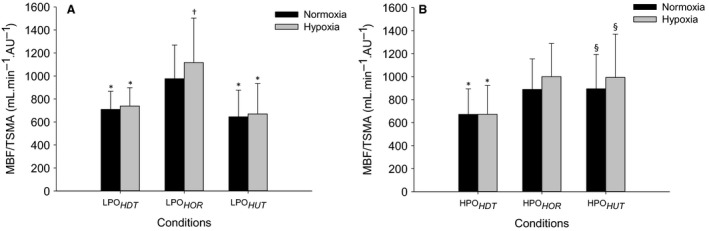
Normalized muscle blood flow by triceps surae Electromyography activity (MBF/TSMA) during plantar flexion exercise in lower (A) and higher power outputs (B). Values are the mean ± SD. See Figure 1 for symbols and abbreviations.

Muscle blood flow normalized by triceps surae muscle activity (MBF/TSMA) revealed significant main effects of body position and F_I_O_2_ in lower power output and a main effect of body position for MBF/TSMA in higher power output. The post hoc analysis indicated that at lower power output, MBF/TSMA was higher in LPO_*HOR*_ compared to LPO_*HDT*_ and LPO_*HUT*_ (*P* < 0.05) with no differences between LPO_*HDT*_ and LPO_*HUT*_ in both normoxia and hypoxia (Fig. 4A). From normoxia to hypoxia at lower power output, MBF/TSMA was higher in hypoxia than normoxia only within LPO_*HOR*_ (*P* < 0.05, Fig. 4A). At the higher power output, MBF/TSMA was lower in HPO_*HDT*_ than HPO_*HOR*_ and HPO_*HUT*_ (*P* < 0.05) with no differences between HPO_*HOR*_ and HPO_*HUT*_ with no differences between normoxia and hypoxia (Fig. 4B).

## Discussion

The delivery of O_2_ to the working muscle at any metabolic demand is affected by gravitational effects on muscle perfusion. This study confirmed previous results (Villar and Hughson 1985; Walker et al. 2007) for submaximal exercise that MBF was higher in the horizontal compared to head‐up or head‐down tilt positions. The adjustment of MBF was accomplished by graded responses of vascular conductance where the vasodilation was greater in HDT than in HOR than in HUT. New results from this study revealed that with the challenge of hypoxia that additional adaptations occur and that at lower power outputs VC had sufficient reserve so that it could increase to meet the metabolic demands, but at higher power outputs in head‐down tilt position, in support of the hypothesis, VC reached its upper functional limit near 100% of peak VC. The consequence was revealed by an increase in triceps surae muscle EMG activity and by a reduced normalized muscle blood flow by triceps surae muscle activity in head‐down tilt being consistent with challenged aerobic metabolism, greater increase in the relative stress of the exercise challenge and the potential for an earlier onset of muscle fatigue.

To achieve the same work rate and metabolic rate in the lower and higher power outputs, the loads were adjusted accordingly to reach the same cable tensions in all body positions. The muscle electromyography monitoring ensured work rates equivalence. Therefore, the changes in VC, MBF, and DO_2est_ reflected vascular regulatory factors that are determined by both metabolic rate and postural effects.

The overall pattern of muscle blood flow in this study was consistent with our previous investigation during normoxia (Villar and Hughson 1985). This was anticipated as the subjects of the previous study returned for this new study of the effects of hypoxia. For the lower power output in head‐up tilt position (LPO_*HUT*_), vascular conductance, muscle blood flow, and triceps surae EMG activity responses did not change significantly, whereas estimated O_2_ delivery decreased ~12% in hypoxia in comparison with normoxia (Table 1, Fig. 3A). This blood flow response is consistent with previous observations during upright seated two‐legged kicking exercise supporting the notion of a reserve in the ability to extract O_2_ to meet the aerobic metabolic demand (MacDonald et al. 2000). With the greater muscle perfusion pressure in LPO_*HUT*_ there was <~20% of the maximal vasodilatory capacity (VC_*peak*_ = 7.0 ± 1.9 mL·min^−1^·mmHg^−1^) in both gas conditions (1.0 ± 0.4 mL·min^−1^·mmHg^−1^ and 1.0 ± 0.4 mL·min^−1^·mmHg^−1^, respectively) to achieve steady‐state muscle blood flow (Fig. 2A).

In contrast, when the reduced muscle perfusion pressure during LPO_*HDT*_ was combined with lower F_I_O_2_, there was an increase in vascular conductance from 5.5 ± 1.6 mL·min^−1^·mmHg^−1^ representing ~75% maximal vasodilatory capacity in normoxia (VC_*peak*_ = 7.1 ± 1.8 mL·min^−1^·mmHg^−1^) to 6.2 ± 1.8 mL·min^−1^·mmHg^−1^ in hypoxia which represented ~85% maximal vasodilatory capacity (VC_*peak*_ = 7.1 ± 1.8 mL·min^−1^·mmHg^−1^) (Fig. 2A). This increased in muscle blood flow (~14%) so that the estimated O_2_ delivery to the exercising muscles was greater than in the same position with normoxia (Table 1). Although, no functional limitation was evident in the recruitment of vascular conductance in LPO_*HDT*_ under hypoxia, the increase in muscle blood flow and estimated O_2_ delivery was associated with a significant increase in the triceps surae muscle activity (~9%) in this condition (Fig.3A). This muscle activity response was assumed to reflect a greater metabolic stress, possibly due to regional limitations in blood flow distribution, suggesting fatigue of some muscle fibers, which could lead to early onset of muscle fatigue even at lower power output. At the cessation of hypoxic exercise in LPO_*HDT*_, muscle blood flow overshoot (Fig. 1A) indicated that even at lower power output, blood flow was to some extent restricted by the muscle contraction. This can be observed in the estimated O_2_ delivery and vascular conductance plots (Fig. 1, B and C, respectively).

Consistent with the LPO_*HOR*_ and LPO_*HUT*_ exercises, vascular conductance, muscle blood flow, and estimated O_2_ delivery were not different during HPO_*HOR*_ and HPO_*HUT*_ between normoxia and hypoxia (Table 1). The results for upright exercise are in agreement with previous studies using one or two‐legged knee extension submaximal exercise (Rowell et al. 1986; Koskolou et al. 1997; Roach et al. 1999; Gonzalez‐Alonso et al. 2001), submaximal handgrip exercise (Wilkins et al. 2006) or calf muscle exercise (Donnelly and Green 2013). During the HPO_*HUT*_ condition, the recruitment of vascular conductance required only ~30% maximal vasodilatory capacity (VC_*peak*_ = 7.0 ± 1.9 mL·min^−1^·mmHg^−1^) in normoxia (1.8 ± 0.5 mL·min^−1^·mmHg^−1^) and hypoxia (1.9 ± 0.6 mL·min^−1^·mmHg^−1^ (Fig. 2B). In HPO_*HUT*_ as in the HPO_*HOR*_, the triceps surae EMG activity was not different from normoxia to hypoxia (Fig. 2B), indicating adequate O_2_ delivery.

At higher power output exercise in head‐down tilt position (HPO_*HDT*_) under altered F_I_O_2_, increased DO_2*est*_ to the working muscles was accomplished by increases in vascular conductance (~14%) and muscle blood flow (~19%) (Table 1). The vascular conductance requirement in HPO_*HDT*_ reached 6.2 ± 1.5 mL·min^−1^·mmHg^−1^ representing ~90% maximal vasodilatory capacity in normoxia (VC_*peak*_ = 7.1 ± 1.8 mL·min^−1^·mmHg^−1^), as previously reported (Villar and Hughson 1985), and increased even further during hypoxia reaching 7.1 ± 1.6 mL·min^−1^·mmHg^−1^ which represented ~100% maximal vasodilatory capacity (VC_*peak*_ = 7.1 ± 1.8 mL·min^−1^·mmHg^−1^) (Fig. 2B). This result indicated that under reduced muscle perfusion pressure with reduced F_I_O_2_, there was a functional limitation to the recruitment of vascular conductance restricting the increase in muscle blood flow leading to a limitation in O_2_ delivery to the working muscles. Interestingly, the absolute values of MBF and DO_2*est*_ in HPO_*HDT*_ were slightly, but not significantly, greater than in HPO_*HUT*_, which could be explained by the higher muscle activity requiring slightly more blood flow and O_2_ delivery to the working muscles to match the metabolic demand imposed by the exercise. In spite of this, a limitation in the ability to supply adequate O_2_ for aerobic energy production in HPO_*HDT*_ was reflected by the increased tripceps surae muscle activity in hypoxia (~20%) compared to normoxia (Fig. 3B) as well as by the reduced normalized muscle blood flow by triceps surae muscle activity response (Fig. 4B).

Higher muscle activity in hypoxia compared with normoxia has been observed in previous studies (Amann et al. 2006). The elevated muscle activity combined with the restricted MBF and DO_2*est*_ responses in HPO_*HDT*_ under hypoxia suggested that greater muscle fiber recruitment was required to maintain power output (Amann et al. 2006; Yasuda et al. 2009), providing evidence of muscle fatigue as a consequence of the metabolic imbalance in HPO_*HDT*_ (Villar and Hughson 1985; Amann and Calbet 2008). During hypoxia in HPO_*HDT*_, the reduced normalized muscle blood flow to triceps surae muscle activity is also an indication of muscle blood flow and O_2_ delivery inadequacy consistent with developing muscle fatigue (Amann and Calbet 2008) and the potential for performance failure (Villar and Hughson 1985) that might reflect regional or microlevel limitations in muscle perfusion in the HDT position when compared to HPO_*HUT*_. At the cessation of exercise under hypoxia, the observed overshoot of muscle blood flow in the HPO_*HDT*_ condition (Fig. 1D) might have reflected relative blood flow deficiency caused by the impediment related to muscle contraction (Villar and Hughson 1985) that added to the effects of lower perfusion pressure.

In conditions with elevated muscle perfusion pressure, VC response was blunted due to the effects of gravity resulting in activation of the myogenic reflex (Villar and Hughson 1985; Imadojemu et al. 2001; Toska and Walloe 2002) and the venoarteriolar reflex (Henriksen and Sejrsen 1977; Villar and Hughson 1985; Tschakovsky and Hughson 2000) resulting in vasoconstriction as well as the activation of the arterial baroreflex increasing sympathetic vasoconstriction (Villar and Hughson 1985; Toska and Walloe 2002; Cui et al. 2003). Adding hypoxia on top of sympathetic vasoconstriction and myogenic components did not result in significant changes in VC compared to normoxia. The interaction between mechanical factors associated with the perfusion pressure and vasodilation in less extent were able to match the metabolic demand despite lower O_2_ availability in hypoxia in both exercise intensities. The absence of overshoot at the cessation of exercise in the head‐up tilt body position conditions support that muscle contraction did not impede muscle blood flow (Fig. 1, A and D).

The increases in vascular conductance from normoxic to hypoxic exercise with constant work rate in head‐down tilt position could be explained by vasodilatory mechanisms (Donnelly and Green 2013). In HDT position, the reduction in muscle perfusion pressure due to gravity promoted an increase in VC due to vascular smooth muscle relaxation (Villar and Hughson 1985), removal of the venoarteriolar reflex (Villar and Hughson 1985; Rowell 1993; Sheriff et al. 2007), reduced baroreflex activity (Villar and Hughson 1985; Toska et al. 1994) and endothelial factors (Shoemaker et al. 1997) resulting in vasodilation. Exercise released vasoactive factors also contributing to vasodilation (Villar and Hughson 1985). Adding hypoxia caused a reduction in arterial O_2_ saturation, arterial O_2_ content and arterial O_2_ partial pressure. Such reductions affect O_2_ availability with consequent decrease in the cellular O_2_ partial pressure stimulating further vasodilatory response (Calbet 2000). The reduced O_2_ is probably sensed by the red blood cells stimulating the release of ATP from the erythrocytes mediating vasodilatory responses (Ellsworth et al. 1995) via nitric oxide and/or endothelium‐derived‐hyperpolarization‐factor and/or release of nitric oxide from S‐nitrosohemoglobin due to hemoglobin deoxygenation relaxing the vascular smooth muscle (Jia et al. 1996; Stamler et al. 1997; Calbet 2000). The accumulation of adenosine during hypoxia could activate the adenosine‐sensitive potassium channels enhancing the release of potassium from the active muscle fiber and elevating the interstitial potassium concentrations (Allen et al. 2008). The adenosine monophosphate‐activated protein kinase is probably activated in hypoxic conditions stimulating the release of nitric oxide and prostaglandins relaxing the vascular smooth muscle (Towler and Hardie 2007; Fisslthaler and Fleming 2009). Other vasoactive factors released during exercise in hypoxia, such as carbon dioxide, hydrogen ions and lactate might also contribute to vasodilatory responses (Calbet 2000).

In summary, muscle blood flow adjustment was achieved due to graded recruitment of vascular conductance with greater vasodilatory responses in HDT and lesser vasodilatory responses in HUT. Under hypoxia at lower power outputs, VC had sufficient reserve to promote the additional adaptations required to meet the metabolic demands of the exercise challenge regarding of body position. However, during lower power output in head‐down tilt position under hypoxia, despite no apparent functional limitation in the vascular conductance recruitment, the rise in muscle blood flow to maintain O_2_ delivery was associated with an increase in triceps surae muscle activity, indicating greater metabolic stress. At the higher power output in HDT, the ability to promote increases in vasodilation to meet the metabolic demands was compromised because vascular conductance recruitment reached its upper functional limit (~100 VC_*peak*_). As a consequence, triceps surae muscle activity increased and the muscle blood flow normalized to triceps surae muscle activity was reduced in HDT. These responses in HDT are consistent with challenged aerobic metabolism contributing to the increase in muscle fiber recruitment with the potential for earlier onset of muscle fatigue.

## Conflict of Interest

None declared.
